# TAS-102 Monotherapy and Combination Therapy with Bevacizumab for Metastatic Colorectal Cancer

**DOI:** 10.1155/2021/4014601

**Published:** 2021-12-20

**Authors:** Cheng-Jiang Liu, Ting Hu, Ping Shao, Wu-Yang Chu, Yu Cao, Feng Zhang

**Affiliations:** ^1^Department of General Medicine, Affiliated Anqing First People's Hospital of Anhui Medical University, 246000, China; ^2^Department of General Practice, Anqing Municipal Hospital of Anhui Medical University, 246000, China; ^3^Department of General Surgery, Tongren Municipal People's Hospital of Guizhou Medical University (GMU), 554300, China

## Abstract

**Objective:**

To evaluate the effectiveness and safety of TAS-102 monotherapy and combination therapy with bevacizumab in the treatment of metastatic colorectal cancer.

**Methods:**

The PubMed, Web of Science, MEDLINE, and Cochrane Library databases were searched for the literature on TAS-102 treatment of metastatic colorectal cancer. Extracted data include median overall survival (mOS), median progression-free survival (mPFS), and the incidence of adverse events for meta-analysis.

**Results:**

Our study found that the mOS of patients treated with TAS-102 monotherapy was 6.95 (95% CI: 6.26-7.72) months and the mPFS was 2.53 (95% CI: 2.31-2.78) months. The mOS in patients treated by TAS-102 combined with bevacizumab was 10.41 (95% CI: 8.40-12.89) months, and the mPFS is 4.35 (95% CI: 3.05-6.20) months. In the control experiment, the patients' mOS and mPFS were improved. TAS-102+B vs. TAS-102 (OR = 0.41, 95% CI: 0.18-0.93; OR = 0.72, 95% CI: 0.63-0.83) and TAS-102 vs. placebo (OR = 0.44, 95% CI: 0.29-0.67; OR = 0.51, 95% CI: 0.42-0.62) were studied to actively prevent the occurrence of neutropenia, leukopenia, febrile neutropenia, anemia, and vomiting.

**Conclusion:**

TAS-102 monotherapy and combination therapy with bevacizumab can significantly improve the survival of patients and prevent specific adverse events from happening.

## 1. Introduction

By 2020, it is estimated that colorectal cancer is the cause of 935,000 cancer-related deaths worldwide, accounting for 9% of all cancer deaths [1]. In the initial diagnosis, approximately 25% of colorectal cancer patients have concurrent metastatic disease, and more than half of the patients are diagnosed as metastases [2, 3]. Despite advances in the treatment of metastatic CRC (metastatic colorectal cancer), the survival rate is still poor. And the expected survival period without effective drug treatment is about 6 months [4–6].

TAS-102 (trifluridine/tipiracil) is an oral anticancer drug containing a thymidine analogue (trifluridine). It is composed of active cytotoxic component FTD and effective thymidine phosphorylase inhibitor TPI hydrochloride. The molar ratio is 1 : 0.5 [5]. FTD is the active cytotoxic component of the drug. TPI can prevent thymidine phosphorylase from rapidly degrading FTD into the inactive form [7, 8]. FTD/TPI is established as the third-line treatment for metastatic colorectal cancer. According to the results of the international phase III RECOURSE study, the study reported the significant benefits of FTD/TPI compared with placebo in terms of overall survival (OS) and acceptable security conditions [9, 10].

The efficacy and safety of FTD/TPI monotherapy in adults with refractory mCRC was first demonstrated in a Japanese phase II trial by Yoshino et al. [5] and later in the pivotal phase III RECOURSE trial [4]. In these two studies, TAS-102 showed good effectiveness, significantly improving median overall survival (mOS) and median progression-free survival (mPFS). TAS-102 combined with bevacizumab had good effectiveness in the treatment of metastatic colorectal cancer, while reducing the incidence of adverse events [11].

Regorafenib and TAS-102 are both considered new treatment options for salvage-line therapy. A meta-analysis showed similar effectiveness of the two drugs, but the occurrence of adverse events may be different [12]. The main goal of clinical trials is to establish the effectiveness and safety of the drug in a carefully selected group of patients. However, there are still differences from real-world applications. The actual application of TAS-102 needs more attention. This study conducted a meta-analysis of clinical trials in the practical application of TAS-102 and compared the safety and effectiveness of drugs in controlled trials and uncontrolled trials.

## 2. Materials and Methods

### 2.1. Search Strategy

PubMed, MEDLINE, Web of Science, and Cochrane databases were searched for eligible publications. The following keywords were used: “metastatic colorectal cancer” AND “TAS-102” OR “FTD/TPI.” There is no time limit for searching until the final search date on May 31, 2021. In addition, the reference list of applicable studies was manually checked for inclusion in other articles. Two researchers jointly completed this search process.

### 2.2. Inclusion and Exclusion

Inclusion criteria are as follows:

1. Patients who participated in the study and who were diagnosed with metastatic colorectal cancer
2. Clinical trials or prospective/retrospective cohort series studies
3. TAS-102 monotherapy or combined therapy with bevacizumab
4. Studies that reported the prognosis of patients after receiving treatment, with at least one of mOS and mPFS or objective response rate (ORR), disease control rate (DCR), and adverse drug reaction (ADR)

Exclusion criteria are as follows:

1. Negative diagnosis or diagnosis mixed with other influential diseases
2. Therapies that included other biological agents or chemotherapy
3. Inconsistent patient baseline data
4. Unobtained full-text articles or unavailable data
5. Animal experiments, reviews, abstracts, reviews, and reports

### 2.3. Data Extraction and Quality Assessment

Two researchers independently extracted relevant information from each study: first author, year of publication, demographic characteristics of participants including age and gender, ECOG performance status, (K) RAS status, grouping scheme, sample size, median OS, median PFS, HR, and the incidence of grade ≥ 3 AEs. We downloaded the full text. If in doubt, ask the original author for help. The Newcastle-Ottawa Quality Assessment Scale is used to evaluate the quality of the included controlled trials. The total score is 9 points, and scores above 5 are included in the meta-analysis. However, for the included one-arm experiment, the first 8 items of the MINORS item were selected for quality evaluation. Each item is 2 points, and the total score is 16 points, and studies with 10 points or more enter our research.

### 2.4. Statistical Analysis

Based on the recommendations of the Cochrane Collaboration, we conducted quantitative synthesis of the indicators included in the study. If *I*^2^ ≤ 50% and *P* > 0.01, heterogeneity was considered to exist, and then a fixed-effects model would be implemented; otherwise, a random-effects model would be performed [13]. If the data were accurate, subgroup analysis based on baseline, interventions, and comparators and/or sensitivity analysis that eliminated studies one by one would be conducted, if appropriate, to explore the source of heterogeneity. Otherwise, we would interpret the result carefully. The small sample effect size and publication bias were detected by funnel plots and statistical tests, respectively [14].

## 3. Results

### 3.1. The Characteristics of the Included Studies

**Figure 1:**
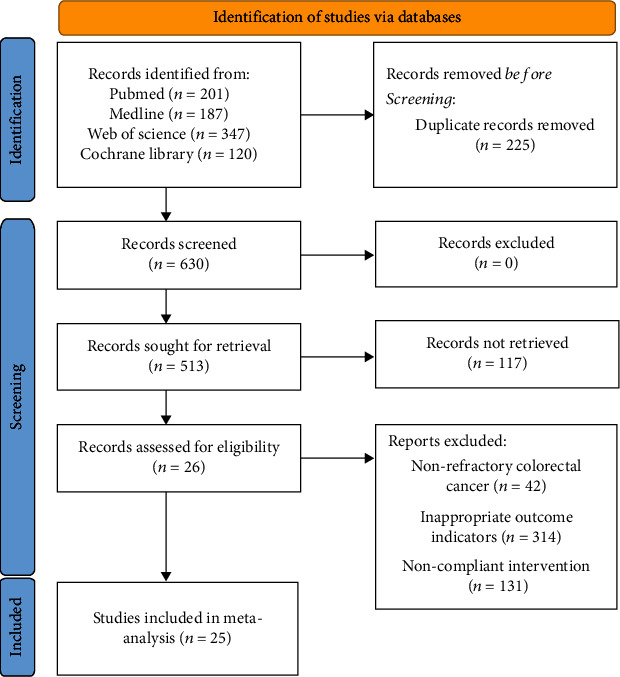
Flow diagram showing the search and screening process.

**Figure 2:**
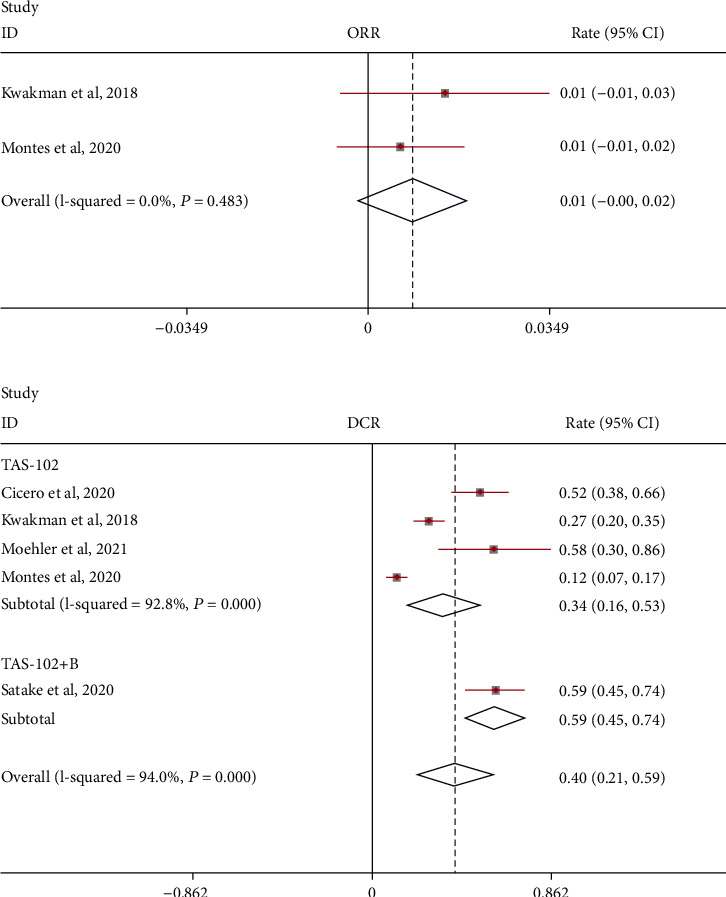
Objective response rate (ORR) and disease control rate (DCR) of TAS-102 monotherapy or combination therapy with bevacizumab for metastatic colorectal cancer.

**Figure 3:**
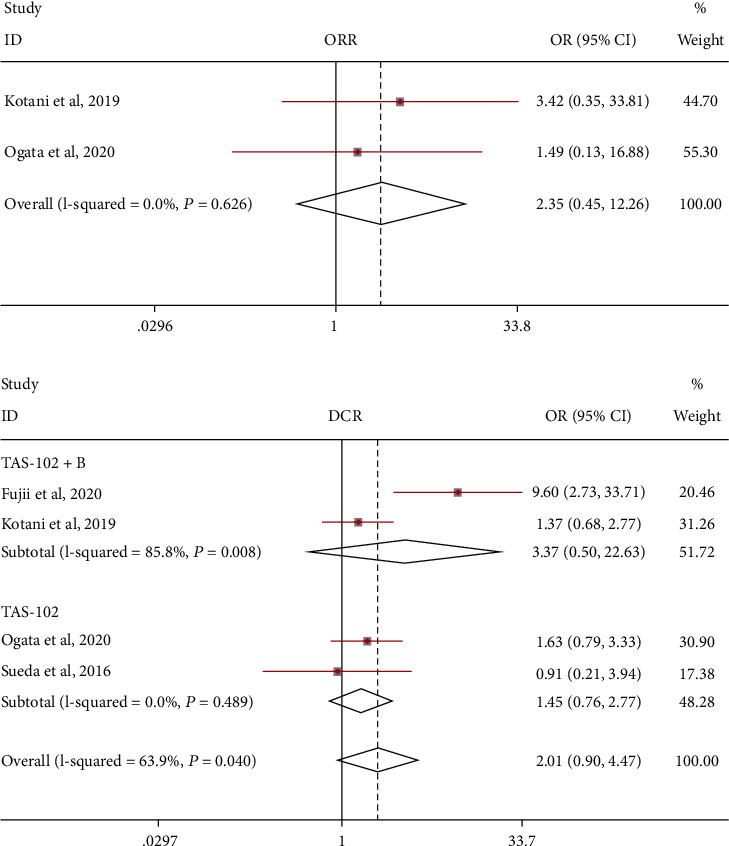
Objective response rate (ORR) and disease control rate (DCR) of those treated with TAS-102 monotherapy or combination therapy with bevacizumab for metastatic colorectal cancer.

855 studies were retrieved. Two investigators screened and included 25 studies [4, 5, 9–11, 15–34]. 14 controlled experiments and 11 single-arm experiments were included. The average age of 3780 participants was over 50 years old. The intervention methods are TAS-102 alone or combined with bevacizumab, and the control is regorafenib (REG) or placebo. The search and screening process is described in Figure 1. All studies included in this study were based on moderate- to high-quality evidence.  Table 1 provides a brief description of these 25 studies. In the included studies, the score of the controlled experiment was above 5, and the score of the uncontrolled experiment was above 10. The quality of the literature can support the meta-analysis. Tables 2–5 and Figures 2 and Figure 3 summarize the literature quality evaluation situation. The registration number of this study in PROSPERO is CRD42021265697.

### 3.2. Effectiveness and Safety of Uncontrolled Clinical Trials

**Figure 4:**
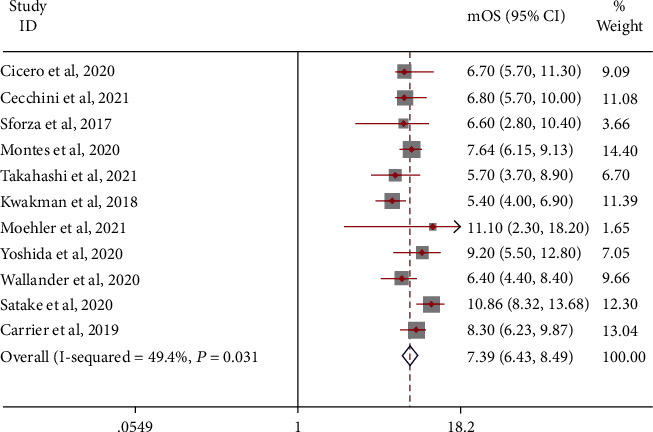
mOS in patients with metastatic colorectal cancer treated with TAS-102 monotherapy or combination therapy with bevacizumab.

Pooling the PFS data from 11 uncontrolled clinical trials revealed that the mOS of patients with metastatic colorectal cancer was 7.39 (95% CI: 6.43-8.49) months with a random-effects model (*I*^2^ = 49.4%, *P* = 0.031; Figure 4). A fixed-effects model was used, and the results were stable (mOS = 7.50, 95% CI: 6.84-8.22 months). Subgroup analysis showed that the mOS of TAS-102 combined with bevacizumab treatment may be higher: TAS-102+B: mOS = 10.41 (95% CI: 8.40-12.89) months and TAS-102: mOS = 6.95 (95% CI: 6.26-7.72) months. A sensitivity analysis that eliminated studies one by one did not detect abnormalities. The funnel chart and Begg's test (Egger's test) show that there is no publication bias.

**Figure 5:**
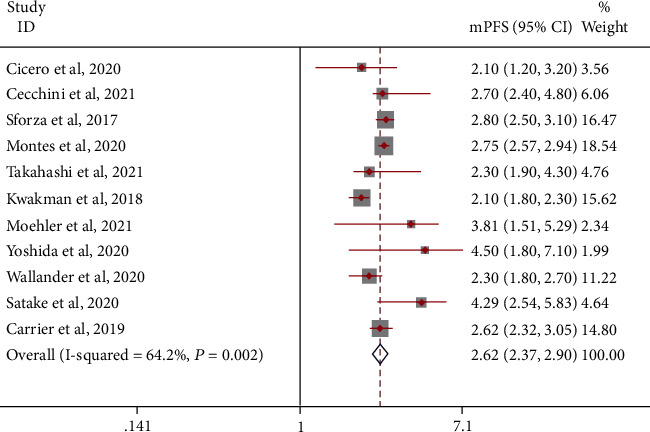
mPFS in patients with metastatic colorectal cancer treated with TAS-102 monotherapy or combination therapy with bevacizumab.

Similarly, the mPFS of patients was 2.62 (95% CI: 2.37-2.90) months. A random-effects model was used (*I*^2^ = 64.2%; Figure 5). A fixed-effects model was used, and the results were stable (mPFS = 2.63, 95% CI: 2.51-2.75 months). Subgroup analysis showed that the mPFS of TAS-102 combined with bevacizumab treatment may be higher: TAS-102+B: mPFS = 4.35 (95% CI: 3.05-6.20) months and TAS-102: mPFS = 2.53 (95% CI: 2.31-2.78) months. A sensitivity analysis that precluded studies one by one did not detect abnormalities. The funnel chart and Begg's test (Egger's test) show that there is no publication bias.

There is no description of the results of the combination of TAS-102 and bevacizumab in the treatment of metastatic colorectal cancer in this study. We use a random-effects model to analyze the objective response rate (ORR) and disease control rate (DCR) (Figure 2). The objective response rate does not seem to be significant and meaningful compared with the disease control rate:ORR = 0.01 (95% CI: -0.00-0.02) and DCR = 0.40 (95% CI: 0.21-0.59). Subgroup analysis showed that the DCR of TAS-102 combined with bevacizumab treatment may be higher: TAS-102+B: DCR = 0.59 (95% CI: 0.45-0.74) and TAS-102: DCR = 0.34 (95% CI: 0.16-0.53).

Grade ≥ 3 adverse events caused by TAS-102 monotherapy or combination therapy with bevacizumab are mainly leukopenia (0.06 and 0.47, respectively), neutropenia (0.30 and 0.10, respectively), decreased appetite (0.10 and 0.06, respectively), and fever (0.10 and 0.05, respectively). The incidence of other hematological or nonhematological adverse events did not reach 0.1. It was worth noting that the combination therapy of TAS-102 and bevacizumab led to multiple grades of adverse events including anemia, thrombocytopenia, vomiting, nausea, asthenia, decreased appetite, diarrhea, fever, and neutropenia.

### 3.3. Effectiveness and Safety of Controlled Clinical Trials

**Figure 6:**
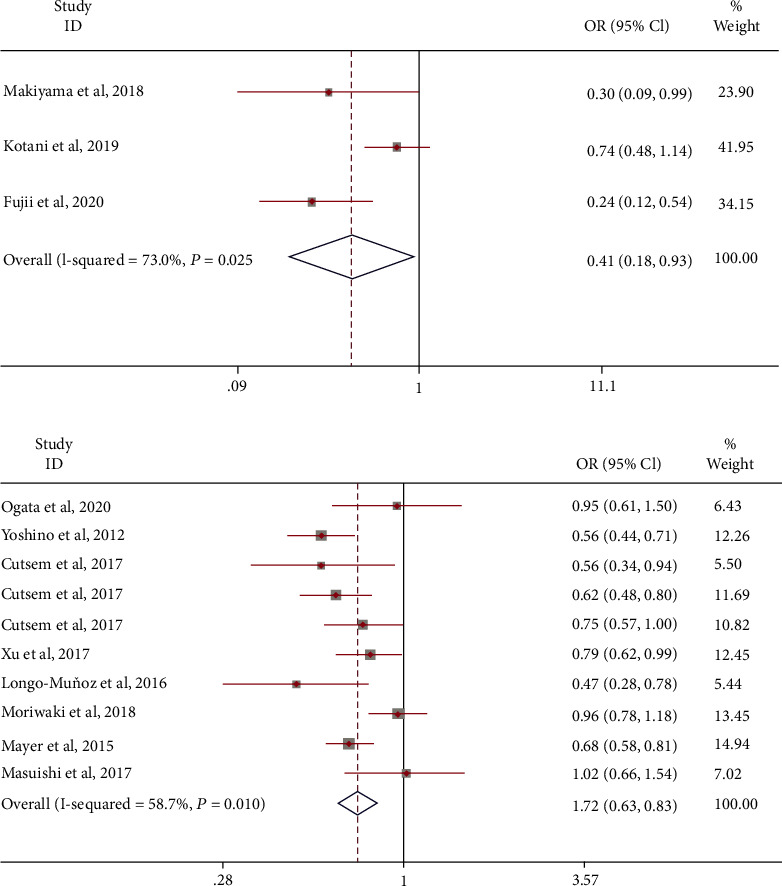
The odds ratio of mOS treated with combination therapy with bevacizumab or TAS-102 monotherapy as the experiment group.

16 controlled clinical trials were included and divided into two designs (TAS-102+B vs. TAS-102 and TAS-102 vs. placebo). Under the first scheme, compared with the control group, the mOS was improved, and the risk ratio of death was 0.41 (95% CI: 0.18-0.93). A random-effects model was used (*I*^2^ = 73.0%; Figure 6). Similarly, the mOS death hazard ratio in the second scheme was 0.72 (95% CI: 0.63-0.83). A random-effects model was used (*I*^2^ = 58.7%; Figure 6). A fixed-effects model was used, and the results were stable. Sensitivity analysis that eliminated studies one by one did not detect abnormalities. The funnel chart and Begg's test (Egger's test) show that there is no publication bias.

**Figure 7:**
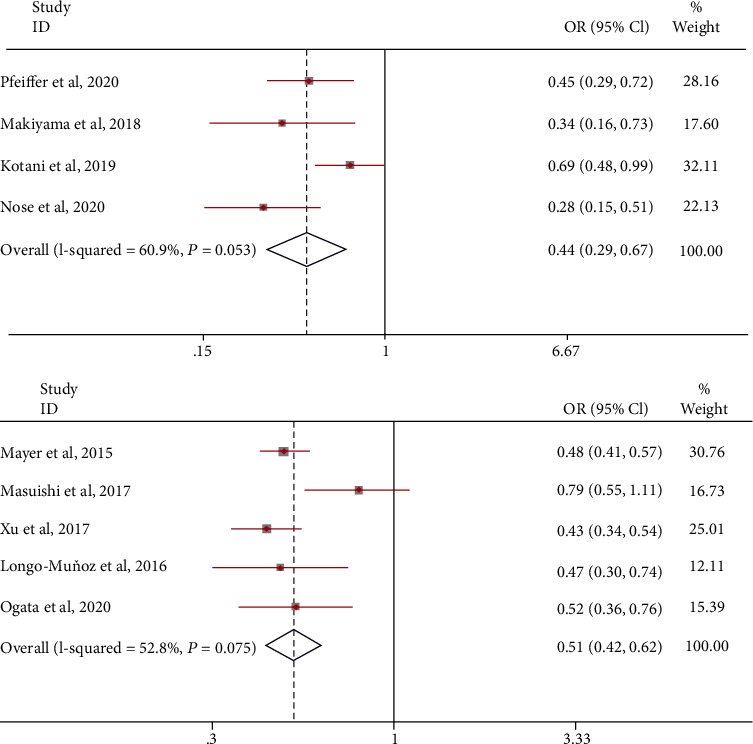
The odds ratio of mPFS treated with combination therapy with bevacizumab or TAS-102 monotherapy as the experiment group.

Under the first scheme, compared with the control group, the mPFS was improved, and the risk ratio of death was 0.44 (95% CI: 0.29-0.67). A random-effects model was used (*I*^2^ = 60.9%; Figure 7). A fixed-effects model was used, and the results were stable. Similarly, the mPFS death hazard ratio in the second scheme was 0.51 (95% CI: 0.42-0.62). A random-effects model was used (*I*^2^ = 52.8%; Figure 7). A sensitivity analysis that eliminated studies one by one did not detect abnormalities. The funnel chart and Begg's test (Egger's test) show that there is no publication bias.

We separately analyzed the objective response rate (ORR) and disease control rate (DCR) of TAS-102 monotherapy versus placebo for metastatic colorectal cancer (Figure 3). However, ORR and DCR were, respectively, comparable in the TAS-102 monotherapy arm and placebo arm [OR = 2.35 (95% CI: 0.45-12.26), OR = 1.45 (95% CI: 0.76-2.77)]. Similarly, the combination of TAS-102 and bevacizumab was comparable with TAS-102 monotherapy in DCR [OR = 3.37 (95% CI: 0.50-22.63)].

Compared with placebo, grade ≥ 3 adverse events caused by TAS-102 may be more extensive and serious (Table 5), for instance, vomiting [OR = 3.72 (95% CI: 1.21-11.43)], neutropenia [OR = 32.40 (95% CI: 12.88-81.52)], anemia [OR = 4.38 (95% CI: 2.78-6.89)], leukopenia [OR = 24.16 (95% CI: 6.12-95.34)], and febrile neutropenia [OR = 7.71 (95% CI: 2.11-28.16)]. The same situation also occurred in any grade of adverse events. However, we only found that bevacizumab combination therapy can increase the occurrence of neutropenia [OR = 2.37 (95% CI: 1.17-4.77)].

## 4. Discussion

Almost 55% of colorectal cancer cases worldwide occur in more developed countries. Its incidence continues to rise in developing countries [35]. As with most cancer types, surgery is the main treatment method. For metastatic cancer, cytotoxic methods, such as neoadjuvant therapy and adjuvant therapy, are used before or after it. The main treatment options include fluoropyrimidine, oxaliplatin, and irinotecan. TAS-102 is an anticancer drug that has entered people's field of vision in recent years. Because of its excellent clinical efficacy and safety, it is often added to the treatment of colorectal cancer and gastric cancer in the middle and late stages and anticancer treatment programs for metastatic tumors.

Our study found that the mOS of patients treated with TAS-102 was 7.74 (95% CI: 6.09-9.85) months and the mPFS was 2.91 (95% CI: 2.38-3.57) months. The mOS in patients treated by TAS-102 combined with bevacizumab is 10.41 (95% CI: 8.40-12.89) months, and the mPFS is 4.35 (95% CI: 3.05-6.20) months. Combination therapy may have better effectiveness. As the current targeted drug for the treatment of metastatic colorectal cancer, it is a humanized monoclonal antibody against vascular endothelial growth factor (VEGF), which plays an antitumor effect by blocking the formation of tumor blood vessels and regulating the immune function of patients [36]. In 2004, the FDA approved bevacizumab combined with chemotherapy drugs as the first-line treatment for mCRC. A study showed that bevacizumab combined with first-line chemotherapy for metastatic colorectal cancer can significantly prolong the survival and PFS of patients with mCRC, improve the quality of life, increase the resectable rate of metastases, and improve the survival outcome of patients with mCRC [37, 38]. The number of adverse events has also been significantly reduced.

Although uncontrolled trials can observe the survival of patients, they cannot specify the improvement in survival. We included 16 studies that included two controlled protocols (TAS-102+B vs. TAS-102 and TAS-102 vs. placebo). In either scenario, we found a significant increase in mOS and mPFS. Surprisingly, we found that TAS-102 combined with bevacizumab will increase the incidence of grade ≥ 3 AEs (OR = 2.19, 95% CI: 1.40-3.44) compared to TAS-102 alone. The safety of bevacizumab is worthy of further consideration. This indicates that clinicians need to make careful decisions when making treatment options for patients with metastatic colorectal cancer, considering the patient's tolerance to anticancer drugs.

It is necessary to optimize the design plan when evaluating the efficacy of new drugs. Randomized controlled trials such as RECOURSE and TERRA are conducted in homogeneous populations, which can minimize the risk of bias [12]. In the current study, we have included real observational studies aimed at evaluating the effectiveness of a relatively small homogeneous population. These studies have the shortcomings of nonrandomized controlled studies. The studies we included have controlled and uncontrolled experiments. And the demographic characteristics and disease manifestations of the participants in the experiment are also quite different. This will actually affect the accuracy of our final results. Therefore, more rigorous and appropriate randomized controlled experiments need to be proposed. The published meta-analysis of TAS-102 involves the comparison of the effectiveness and safety of multiple therapeutic drugs [12, 39–43]. Regorafenib, TAS-102, fruquintinib, panitumumab, and cetuximab are recommended single-agent chemotherapy regimens for patients exhibiting disease progression. The safety of these drugs is difficult to assess. But the safety of the drug does affect the confidence of patients in the treatment plan. The most important thing is the improvement of symptoms and the management of side effects [44, 45].

In recent years, people have tried to develop a risk prognostic model for metastatic colorectal cancer [46, 47]. Although these analyses differ in methods and patient populations, ECOG PS, KRAS status, and the number of metastatic sites are common factors in many models. We observed that multiple stratification factors, including KRAS status, may affect the benefit of all patients from TAS-102 treatment but have no effect on the prognostic index. A better prognosis often puts higher requirements on the patient's body tolerance and survival status.

Heterogeneity is often an important factor in measuring the accuracy of meta-analysis results. It seems to be an unavoidable issue in evaluating the two important results of this study (mOS and mPFS). We use a random-effects model and fixed-effects model to mutually verify the final results and finally show that they are trustworthy. We have noticed that the highest proportion of women in the patient population in this study is 59.46% and the highest proportion of mutants in the KRAS status is 67.74%. Perhaps, it is because of this that a higher survival prognosis is obtained (mOS: 22.4 months, mPFS: 9.4 months). The choice of control drugs in controlled clinical trials to evaluate TAS-102 may be an important reason for the heterogeneity between studies. There is a significant difference in mOS between the placebo and the antitumor drug regorafenib. OR was 0.66 (95% CI: 0.59-0.74) and 0.97 (95% CI: 0.82-1.15), respectively. For another indicator (mPFS), there is no change. However, clinicians need to be cautious in their practical application.

This study has proved the good prognosis of TAS-102 monotherapy and combination therapy with bevacizumab for metastatic colorectal cancer. However, the occurrence of grade ≥ 3 AEs and any grade of adverse events is still worthy of attention. Even if it may be due to fewer experiments or a different patient population, it needs to be verified by more rigorous and randomized controlled clinical trials.

## Figures and Tables

**Table 1:** Characteristics of included studies.

Study	Age (years)	Sex (male/female)	ECOG performance status (0/≥1)	KRAS status (wild/mutated)	Methods	Sample	mOS (months)	mPFS (months)
Mayer et al. 2015 [4]	63 (27-82)	326/208	301/233	272/262	TAS-102	534	7.1 (6.5-7.8)	2 (1.9-2.1)
	63 (27-82)	165/101	147/119	131/135	Placebo	266	5.3 (4.6-6.0)	1.7 (1.7-1.8)
Pfeiffer et al. 2020 [15]	64 (57-69)	24/22	23/23	19/27	TAS-102+B	46	NA	4.6 (3.5-6.5)
	67 (58-72)	30/17	15/32	18/29	TAS-102	47	NA	2.6 (1.6-3.5)
Sueda et al. 2016 [16]	66 (44-80)	10/4	1/13	9/5	TAS-102	14	6.3 (3.21-9.93)	1 (0.92-6.39)
	59 (37-83)	12/11	10/13	12/11	REG	23	5.8 (3.7-11.7)	0 (1.64-4.52)
Masuishi et al. 2017 [17]	NA	30/24	NA	21/32	TAS-102	54	6.5 (5.3-8.3)	2.1 (1.8-3.1)
	NA	90/56	NA	78/67	REG	146	6.7 (5.8-7.6)	2.1 (1.8-2.5)
Makiyama et al. 2018 [18]	66 (39-82)	6/5	5/6	NA	TAS-102+B	11	Not reached	5.8
	69 (47-82)	20/13	11/22	NA	TAS-102	33	6.4	1.8
Yoshino et al. 2012 [5]	63 (28-80)	64/48	72/40	54/45	TAS-102	112	9.0 (7.3-11.3)	NA
	62 (39-79)	28/29	35/22	24/26	Placebo	57	6.6 (4.9-8.0)	NA
Cutsem et al. 2017 [9]	60.2 (11.86)	31/33	28/36	35/29	TAS-102	64	6.5	NA
	58.5 (11.02)	18/17	13/22	17/18	Placebo	35	4.3	NA
	61.8 (9.98)	167/104	138/133	123/148	TAS-102	271	NA	NA
	62.1 (10.42)	82/50	68/64	68/64	Placebo	132	NA	NA
	61.9 (10.09)	113/65	128/50	94/84	TAS-102	178	7.8	NA
	62.1 (10.40)	58/30	60/28	40/48	Placebo	88	6.7	NA
Xu et al. 2017 [10]	58 (26-81)	170/101	64/207	172/99	TAS-102	271	7.8 (7.1-8.8)	NA
	56 (24-80)	84/51	30/105	85/50	Placebo	135	7.1 (5.9-8.2)	NA
Longo-Muñoz et al. 2016 [19]	5 (27-81)	48/32	24/56	35/45	TAS-102	80	6.8	2
	5 (39-78)	21/11	11/21	17/15	Placebo	32	4.6	1.7
Moriwaki et al. 2018 [20]	64 (29-86)	197/130	128/199	160/161	TAS-102	327	7.4 (6.6-8.3)	NA
	64 (31-84)	126/97	95/128	88/109	REG	223	7.9 (6.8-9.2)	NA
Kotani et al. 2019 [21]	60 (23-79)	35/25	35/25	28/32	TAS-102+B	60	8.6 (6.9-10.3)	3.7 (2.3-5.1)
	65 (30-80)	42/24	42/24	30/36	TAS-102	66	8.0 (6.7-9.4)	2.2 (1.8-2.6)
Fujii et al. 2020 [11]	67 (50-74)	13/8	NA	10/11	TAS-102+B	21	14.4 (7.9-NA)	NA
	67.5 (59.8-71.2)	16/20	NA	16/20	TAS-102	36	4.5 (3.2-6.5)	NA
Ogata et al. 2020 [22]	68 (40-85)	38/39	35/42	53/24	TAS-102	77	11.4	3.3
	66 (41-81)	30/27	30/27	36/21	REG	57	9.9	2
Nose et al. 2020 [23]	73 (49-90)	16/16	12/20	14/17	TAS-102+B	32	11.7	4.7
	70.5 (43-88)	15/9	7/17	14/10	TAS-102	24	6.3	1.8
Cicero et al. 2020 [24]	78 (70-86)	28/22	18/32	18/22	TAS-102	50	6.7 (5.7-11.3)	2.1 (1.2-3.2)
Cecchini et al. 2021 [25]	NA	NA	NA	NA	TAS-102	41	6.8 (5.7-10)	2.7 (2.4-4.8)
Sforza et al. 2017 [26]	65 (48-82)	31/12	27/16	16/27	TAS-102	43	6.6 (2.8-10.4)	2.8 (2.5-3.1)
Montes et al. 2020 [27]	63 (37-83)	108/52	18/142	57/103	TAS-102	160	7.64 (6.15-9.13)	2.75 (2.57-2.94)
Takahashi et al. 2021 [28]	73 (65-81)	21/9	NA	NA	TAS-102	30	5.7 (3.7-8.9)	2.3 (1.9-4.3)
Kwakman et al. 2018 [29]	62 (30-88)	92/44	46/90	53/83	TAS-102	136	5.4 (4.0-6.9)	2.1 (1.8-2.3)
Moehler et al. 2021 [30]	60 (35-78)	6/6	6/6	NA	TAS-102	12	11.1 (2.3-18.2)	3.81 (1.51-5.29)
Yoshida et al. 2020 [31]	67 (45-78)	20/12	23/9	14/18	TAS-102+B	32	9.2 (5.5-12.8)	4.5 (1.8-7.1)
Wallander et al. 2020 [32]	65 (38-78)	28/20	13/34	17/29	TAS-102	48	6.4 (4.4-8.4)	2.3 (1.8-2.7)
Satake et al. 2020 [33]	69 (33-82)	24/20	25/19	25/19	TAS-102+B	44	10.86 (8.32-13.68)	4.29 (2.54-5.83)
Carries et al. 2019 [34]	65.29 (40-88)	49/35	13/71	31/53	TAS-102	84	8.3 (6.23-9.87)	2.62 (2.32-3.05)

**Table 2:** The Newcastle-Ottawa Quality Assessment Scale for included controlled studies.

Study	Selection of the study groups	Comparability of the groups	Outcome	Total score
Mayer et al. 2015 [4]	⭐⭐⭐⭐	⭐	⭐⭐	7
Pfeiffer et al. 2020 [15]	⭐⭐⭐⭐	⭐	⭐⭐	7
Sueda et al. 2016 [16]	⭐⭐⭐⭐	⭐	⭐⭐	7
Masuishi et al. 2017 [17]	⭐⭐⭐⭐	⭐	⭐⭐	7
Makiyama et al. 2018 [18]	⭐⭐⭐⭐	⭐	⭐⭐	7
Yoshino et al. 2012 [5]	⭐⭐⭐⭐	⭐	⭐⭐	7
Cutsem et al. 2017 [9]	⭐⭐⭐⭐	⭐	⭐⭐	7
Xu et al. 2017 [10]	⭐⭐⭐⭐	⭐⭐	⭐⭐	8
Longo-Muñoz et al. 2016 [19]	⭐⭐⭐⭐	⭐⭐	⭐⭐	8
Moriwaki et al. 2018 [20]	⭐⭐⭐⭐	⭐⭐	⭐⭐	8
Kotani et al. 2019 [21]	⭐⭐⭐⭐	⭐⭐	⭐⭐	8
Fujii et al. 2020 [11]	⭐⭐⭐⭐	⭐	⭐⭐	7
Ogata et al. 2020 [22]	⭐⭐⭐⭐	⭐	⭐⭐	7
Nose et al. 2020 [23]	⭐⭐⭐⭐	⭐⭐	⭐⭐	8

**Table 3:** MINORS quality evaluation for included uncontrolled studies.

Study	Clear purpose	Patient continuity	Data collection	Appropriate endpoint	Objective evaluation endpoint	Adequate follow-up time	Low lost to follow-up rate	Sample size estimation	Total score
Cicero et al. 2020 [24]	2	2	2	2	1	2	1	0	12
Cecchini et al. 2021 [25]	2	2	2	2	1	2	0	0	11
Sforza et al. 2017 [26]	2	2	2	2	1	2	2	0	13
Montes et al. 2020 [27]	2	2	2	2	1	2	1	0	12
Takahashi et al. 2021 [28]	2	2	1	2	1	2	1	0	11
Kwakman et al. 2018 [29]	2	2	2	2	2	2	1	0	13
Moehler et al. 2021 [30]	2	2	1	2	1	1	2	0	11
Yoshida et al. 2020 [31]	2	2	2	2	2	2	2	1	15
Wallander et al. 2020 [32]	2	2	2	2	1	2	2	0	13
Satake et al. 2020 [33]	2	2	2	2	2	2	1	2	15
Carries et al. 2019 [34]	2	2	2	2	1	2	1	0	12

**Table 4:** Meta-analysis results for the occurrence of adverse events in uncontrolled experiments.

Outcomes	Any grade					Grade > 3				
	Methods	Trials	Rate (95% CI)	*I* ^2^	*P*	Methods	Trials	Rate (95% CI)	*I* ^2^	*P*
*Nonhematological*										
Vomiting	TAS-102	5	0.10 (0.04-0.16)	57.60%	<0.001	TAS-102	3	0.02 (-0.00-0.05)	0%	0.059
	TAS-102+B	2	0.21 (0.12-0.30)	0%	<0.001					
Nausea	TAS-102	6	0.27 (0.17-0.38)	81.00%	<0.001	TAS-102	2	0.01 (-0.01-0.04)	0%	0.251
	TAS-102+B	2	0.58 (0.47-0.69)	0%	<0.001	TAS-102+B	2	0.07 (0.01-0.12)	0%	0.021
Asthenia	TAS-102	8	0.36 (0.25-0.47)	86.20%	<0.001	TAS-102	6	0.05 (0.03-0.08)	0%	<0.001
	TAS-102+B	2	0.56 (0.40-0.72)	53.60%	<0.001	TAS-102+B	1	0.03 (-0.03-0.09)		0.31
Decreased appetite	TAS-102	4	0.25 (0.13-0.37)	75.90%	<0.001	TAS-102	1	0.10 (-0.01-0.21)	NA	0.068
	TAS-102+B	1	0.66 (0.49-0.82)	NA	<0.001	TAS-102+B	1	0.06 (-0.02-0.15)	NA	0.144
Diarrhea	TAS-102	7	0.13 (0.07-0.19)	76.00%	<0.001	TAS-102	5	0.06 (0.01-0.12)	75.30%	0.023
	TAS-102+B	2	0.22 (0.12-0.31)	1.90%	<0.001	TAS-102+B	1	0.03 (0.00-0.05)	NA	0.043
Abdominal pain	TAS-102	3	0.17 (0.04-0.30)	72.30%	0.012					
Fever	TAS-102	4	0.06 (0.02-0.10)	0%	0.001	TAS-102	1	0.10 (-0.01-0.21)	NA	0.068
	TAS-102+B	1	0.18 (0.07-0.30)	NA	0.002	TAS-102+B	1	0.05 (-0.02-0.11)	NA	0.148
*Hematological*										
Neutropenia	TAS-102	7	0.55 (0.43-0.67)	84.70%	<0.001	TAS-102	8	0.30 (0.26-0.35)	26.50%	<0.001
	TAS-102+B	2	0.67 (0.57-0.78)	0%	<0.001	TAS-102+B	2	0.10 (0.01-0.20)	47.80%	0.029
Anemia	TAS-102	6	0.49 (0.18-0.80)	98.70%	0.002	TAS-102	8	0.07 (0.05-0.09)	4.50%	<0.001
	TAS-102+B	2	0.89 (0.82-0.96)	0%	<0.001	TAS-102+B	2	0.09 (0.03-0.16)	0%	0.005
Leukopenia	TAS-102	2	0.66 (0.58-0.74)	0%	<0.001	TAS-102	2	0.06 (0.03-0.09)	0%	<0.001
	TAS-102+B	1	0.72 (0.56-0.87)	NA	<0.001	TAS-102+B	1	0.47 (0.30-0.64)	NA	<0.001
Febrile neutropenia	TAS-102	2	0.09 (-0.02-0.21)	74.90%	0.113	TAS-102	3	0.08 (0.02-0.14)	49.70%	0.012
Thrombocytopenia	TAS-102	6	0.26 (0.12-0.39)	93.7%	<0.001	TAS-102	5	0.01 (0.00-0.02)	8.60%	0.014
	TAS-102+B	2	0.37 (0.21-0.53)	0%	<0.001	TAS-102+B	2	0.06 (0.01-0.12)	30.40%	0.022

**Table 5:** Meta-analysis results for the occurrence of adverse events in controlled experiments.

Outcomes	Any grade					Grade > 3				
	Intervention	Trials	OR (95% CI)	*I* ^2^	*P*	Intervention	Trials	OR (95% CI)	*I* ^2^	*P*
*Nonhematological*										
Vomiting	TAS-102	5	2.99 (2.17-4.13)	16.70%	<0.001	TAS-102	5	3.72 (1.21-11.43)	0%	0.022
	TAS-102+B	1	0.53 (0.09-3.03)		0.479					
Nausea	TAS-102	3	3.32 (1.31-4.44)	0%	<0.001	TAS-102	2	1.79 (0.54-5.90)	0%	0.338
	TAS-102+B	2	0.80 (0.36-1.78)	0%	0.59	TAS-102+B				
Asthenia	TAS-102	5	1.45 (1.08-121.96)	55.40%	0.015	TAS-102	8	0.85 (0.58-1.25)	0%	0.4
	TAS-102+B	2	1.43 (0.76-2.66)	0%	0.265	TAS-102+B	2	0.59 (0.11-3.17)	0%	0.534
Decreased appetite	TAS-102	6	1.43 (0.90-2.26)	61.90%	0.127	TAS-102	7	0.88 (0.58-1.32)	0%	0.527
	TAS-102+B	1	0.54 (0.13-2.29)	NA	0.405	TAS-102+B	2	0.17 (0.02-1.42)	0%	0.1
Diarrhea	TAS-102	4	1.63 (0.79-3.37)	55.70%	0.043	TAS-102	3	1.30 (0.13-12.59)	60.60%	0.82
	TAS-102+B	2	0.73 (0.10.5.62)	0%	0.453	TAS-102+B	1	0.14 (0.01-3.02)	NA	0.209
Abdominal pain	TAS-102	2	1.23 (0.86-1.76)	0%	0.256	TAS-102	4	0.57 (0.30-1.06)	0%	0.075
Fever	TAS-102	3	0.42 (0.09-2.02)	86.60%	0.277	TAS-102	2	3.14 (0.54-18.10)	0%	0.201
*Hematological*										
Neutropenia	TAS-102	4	28.21 (1.40-568.32)	96.60%	0.029	TAS-102	9	32.40 (12.88-81.52)	31.00%	<0.001
Anemia	TAS-102+B	1	3.33 (1.10-10.12)	NA	0.034	TAS-102+B	3	2.37 (1.17-4.77)	34.20%	0.016
	TAS-102	3	4.94 (3.11-7.85)	63.50%	<0.001	TAS-102	8	4.38 (2.78-6.89)	26.70%	<0.001
	TAS-102+B	2	0.58 (0.20-1.69)	0%	0.321	TAS-102+B	2	0.61 (0.25-1.48)	0%	0.272
Leukopenia	TAS-102	2	72.00 (42.51-121.95)	0%	<0.001	TAS-102	5	24.16 (6.12-95.34)	14.10%	<0.001
	TAS-102+B	1	1.80 (0.77-4.19)	NA	0.172	TAS-102+B	1	1.54 (0.73-3.24)	NA	0.258
Febrile neutropenia	TAS-102	2	7.83 (0.75-81.26)	17.70%	0.085	TAS-102	5	7.71 (2.11-28.16)	0%	0.002
	TAS-102+B	1	0.42 (0.08-2.25)	NA	0.312	TAS-102+B	1	2.24 (0.20-25.37)	NA	0.514
Thrombocytopenia	TAS-102	4	2.27 (0.51-10.22)	93.10%	0.284	TAS-102	6	1.21 (0.38-3.80)	64.30%	0.749
	TAS-102+B	2	2.17 (0.39-11.91)	41.90%	0.374	TAS-102+B	1	0.74 (0.04-12.49)	NA	0.836
